# Brain Breaks® Physical Activity Solutions in the Classroom and on Attitudes toward Physical Activity: A Randomized Controlled Trial among Primary Students from Eight Countries

**DOI:** 10.3390/ijerph17051666

**Published:** 2020-03-04

**Authors:** Magdalena Mo Ching Mok, Ming-Kai Chin, Agata Korcz, Biljana Popeska, Christopher R. Edginton, Fatma Sacli Uzunoz, Hrvoje Podnar, Dané Coetzee, Luminita Georgescu, Arunas Emeljanovas, Milan Pasic, Govindasamy Balasekaran, Elizabeth Anderson, J. Larry Durstine

**Affiliations:** 1 Graduate Institute of Educational Information and Measurement, National Taichung University of Education, Taichung City 40306, Taiwan; mmcmok@friends.eduhk.hk; 2 Department of Psychology | Assessment Research Centre, The Education University of Hong Kong, 10 Lo Ping Road, Taipo, N.T., Hong Kong; 3 The Foundation for Global Community Health, Henderson, NV 89012, USA; chinmingkai@yahoo.com; 4 Department of Didactics of Physical Activity, Poznan University of Physical Education, Poznan 61-871, Poland; 5 Faculty of Educational Sciences, Goce Delcev University, Stip 2000, Macedonia; biljana.popeska@ugd.edu.mk; 6 Human Performance Center, University of Northern Iowa, 105, Cedar Falls, IA 50614, USA; christopher.edginton@uni.edu; 7 School of Sport Sciences and Technology, Department of Coaching Education, Nevşehir Hacı Bektaş Veli University, 50300 Nevşehir, Turkey; fatmauzunoz5050@gmail.com; 8 Faculty of Kinesiology, University of Zagreb, 10000 Zagreb, Croatia; hrvoje@podnar.com; 9 Physical Activity, Sport and Recreation (PhASRec), Focus Area, School of Human Movement Sciences, Faculty of Health Sciences, North-West University, Mafikeng 2790, South Africa; dane.coetzee@nwu.ac.za; 10 Department of Physical Education and Sport, University of Pitesti, 110040 Pitesti, Romania; kinetopit@yahoo.com; 11 Institute of Health Sciences, Medical Faculty, Vilnius University, LT-10222 Vilnius, Lithuania; arunas.emeljanovas@lsu.lt; 12 Primary School Ivo Andric, 11090 Belgrade, Serbia; pasic_milan@yahoo.com; 13 Department Coaching in Sport, Faculty of Sport, University Union, 11070 Belgrade, Serbia; 14 Physical Education and Sports Science, National Institute of Education, Nanyang Technological University, Singapore 637616, Singapore; govindasamy.b@nie.edu.sg; 15 Department of Exercise Science, University of South Carolina, Columbia, SC 29208, USA; ea2@mailbox.sc.edu (E.A.); ldurstin@mailbox.sc.edu (J.L.D.)

**Keywords:** physical activity, pediatrics, physical fitness, public health, teaching, youth

## Abstract

Classroom-based physical activity (PA) interventions have received considerable attention due to improvements seen in academic achievement, classroom behaviors, and attitude toward PA. The purpose of this study was to evaluate the effectiveness of the Brain Breaks® Physical Activity Solutions in changing children’s attitudes toward PA. Students (*N* = 3036) aged 8–11 years from schools in Croatia, Lithuania, Macedonia, Poland, Romania, Serbia, South Africa, and Turkey were randomly assigned to either a control or an experimental group. The experimental group received Brain Breaks® videos during classroom sessions throughout the four months of intervention. Student attitudes toward PA were measured using the Attitudes toward Physical Activity Scale (APAS) before and after the intervention. Repeated measures ANOVA indicated a time interaction effect for all APAS variables except fitness. Time-by-group interaction effects with different effect sizes were found for most APAS variables, with the greatest gain effect noted in the experimental group for self-efficacy, followed by learning from the videos concerning PA benefits, exercise importance, and enjoyment from engaging in PA. This study provides evidence supporting Brain Breaks® in terms of learning experience, attitudes towards PA, and personal motivation. Using exercise videos is recommended as an interactive, technology-based PA solution that can be easily integrated into the school setting.

## 1. Introduction

Physical inactivity is one of the leading risk factors for death worldwide, while physical activity (PA) provides significant health benefits and contributes to the prevention of non-communicable diseases (NCDs) [1]. Approximately 23% of adults and 81% of adolescents report insufficient daily PA in 2010 [2]. At the same time, physical inactivity is associated with obesity. The Turkey Nutrition and Health Survey—Evaluation of Nutritional Status and Habits Report found that 8.2% of children aged 6–18 years were obese and 14.3% were overweight [3]. Data concerning children from South Africa and Serbia demonstrate increased physical inactivity and obesity [4,5]. Similar trends are seen in other countries participating in the WHO European Childhood Obesity Surveillance Initiative (COSI) [4]. A meta-analysis regarding children from Romania also report similar prevalence rates for being overweight and obesity (23.2–28.3%) [6].

Existing literature emphasizes the positive effects of PA on children’s motor development [7], physical fitness [8], cognition, attention, learning [9,10], academic achievement [11,12,13], and mental health [8]. Unfortunately, PA levels are decreasing for children while childhood health problems rise [14]. Furthermore, most children fail to meet WHO PA recommendations [15]. The World Health Organization (WHO) recommends that children should engage in 60 min of daily moderate-to-vigorous physical activity (dMVPA). Children fail to meet WHO’s PA recommendations as a result of numerous factors related to contemporary ways of living, use of technology [16], crowded school curriculum, and classroom sitting [17]. In support of this premise, Tremblay et al. [17] reported that high sedentary behavior in children is manifested as excessive screen viewing, increased use of digital technology, and is closely related with low PA levels. 

Schools have been identified as important arenas for PA and healthy lifestyle promotion [18], and are recognized as an excellent environment for the implementation of PA interventions because of the access to children, no cost to families [19], controlled environment in the school setting, and significant time children spend in school [20]. Trudeau and Shepard [21] reported that adding more physical education to the school curriculum did not hinder student academic achievement, whereas taking time from physical education programs to allow for more study time did not enhance academic achievement. Thus, new strategies are needed to increase PA opportunities for children during school hours.

Present literature support school-based PA interventions as an effective strategy for improving health outcomes [22] and academic achievement [13,16]. Positive effects are also noted in terms of brain function [11], maintaining student attention [12], and increased PA in school settings [23].

Active breaks during school are effective at improving children’s cognitive function [12,15], academic achievement [11,13], and classroom behavior [24]. Teachers employing such learning strategies prefer activity breaks that are quick and easy to manage, academically oriented, and enjoyable for students [25]. Current studies support technology as being effective at promoting active lifestyles [26]. Different interactive video games, internet platforms, and internet-based PA interventions exist, are attractive to children, and provide opportunities to engage in active games, experience fun [27], stimulate interest, and offer a learning experience with active movement [28,29,30].

One multilevel intervention that combines classroom-based PA and modern technology, while integrating holistic learning among children, is HOPSports Brain Breaks® Physical Activity Solutions [31]. However, studies on the influence of Brain Breaks videos on student attitudes toward PA from diverse countries is lacking. The purpose of this study was to evaluate the effects of implementing Brain Breaks® videos on student attitudes toward PA during a four-month intervention program using students from eight countries. The working hypothesis is regular participation in classroom PA breaks will positively affect student attitudes toward PA.

## 2. Methods

### 2.1. Design and Participants

Primary grade students from Croatia, Lithuania, Macedonia, Poland, Romania, Serbia, South Africa, and Turkey voluntarily participated in the study. The final sample was comprised of 3036 (1496 male, 1540 female) primary students in grades 3, 4, and 5 from 120 classes from 16 schools from the eight countries. The sample subject distribution is found in Table 1 and each country sample distribution is found in the Supplementary Table S1.

A two-group experimental and control pre- and post-test quasi-experimental design was adopted. Classrooms were randomly assigned to either control (49 classrooms comprised 1122 students from 11 schools) or experimental (71 classrooms comprised 1914 students from 14 schools) groups. Whereas all countries were adequately represented in both groups, nuances across countries existed in the way classrooms were assigned to either control or experimental groups given country contextual differences, including class sizes and educational regulations. Romania and South Africa randomly assigned schools (including all classrooms within the schools) to either the control or experimental group. The remaining six countries randomly assigned classrooms (rather than schools) to control or experimental groups.

### 2.2. Intervention: Brain Breaks®

Students in the experimental group performed a series of 3-to-5-minute group activity exercise videos within the classroom during a school day; the videos were provided by HOPSports Brain Breaks® Physical Activity Solutions (http://hopsports.com/what-is-brain-breaks). Each Brain Breaks video provided movement-integrated teaching with motor and fitness skills presented by animated and real-life instructors. Different fundamental movements were presented in the videos, including warm-up exercises, elements from different sports and traditional dances, and traditional or popular music from different countries worldwide. In addition to PA, the content of the video incorporated health and nutrition education, social learning, environmental stewardship, core curricular learning, character development, and exposure to arts and culture. Prior to the intervention, teachers for the experimental group were instructed by trained research assistants in intervention implementation procedures and how to lead exercises. Teachers were provided with online access to the Brain Breaks® administration platform and submitted monthly reports on their video use. Videos were 3–5 minutes in length, presented two times per day, 5 days each week. Students in the control group did not receive any Brain Breaks® interventions and were only given standard teaching and materials. Both groups completed an anonymous 30-minute self-report questionnaire administered by teachers before and after the intervention. The questionnaire was designed to collect data on students’ attitudes toward PA, particularly regarding subjects’ personal estimate of their physical fitness level, self-efficacy, goal orientation, interest toward PA, self-awareness for the importance and benefits of PA, and PA’s contribution in learning about health and holistic development. All testing was completed anonymously using a code designed to match students’ responses at pre- and post-intervention without revealing the student’s identity. 

### 2.3. Measures

The Attitudes toward Physical Activity Scale (APAS) questionnaire was validated and reported earlier [28,29,30,32]. The APAS questionnaire was translated and reviewed for cultural appropriateness, modified when necessary, and translated back to English for verification via independent review in order to ensure reliability and comparability of the data collected. All participating countries went through the same vigorous language and cultural adaptation with a verification process to ensure reliability and validity.

A questionnaire asking for student demographic information regarding subjects’ gender, age, grade level, body weight, and height was completed. The APAS questionnaire containing the seven scales designed to measure students’ attitudes toward PA [33] was administered.

(1) Benefits: A 10-item scale (Cronbach’s alpha = 0.878; McDonald’s omega = 0.879) constructed to measure students’ perceived benefits of PA.
(2) Importance: A 5-item scale (Cronbach’s alpha = 0.800; McDonald’s omega = 0.804) constructed to measure students’ perceived importance of PA.
(3) Learning: An 11-item scale (Cronbach’s alpha = 0.929; McDonald’s omega = 0.928) constructed to measure students’ learning from the videos.
(4) Self-efficacy: A 4-item scale (Cronbach’s alpha = 0.878; McDonald’s omega = 0.878) constructed to measure students’ self-efficacy in selecting video exercises for themselves.
(5) Fun: A 14-item scale (Cronbach’s alpha = 0.920; McDonald’s omega = 0.920) constructed to measure students’ interest in doing PA.
(6) Fitness: An 8-item scale (Cronbach’s alpha = 0.881; McDonald’s omega = 0.881) constructed to measure students’ confidence in their own fitness.
(7) Personal best: A 5-item scale (Cronbach’s alpha = 0.898; McDonald’s omega = 0.898) constructed to measure students’ orientation to their personal best goals when engaging in PA [34].

Response options for the APAS items involved a four-point Likert scale with options of strongly disagree, disagree, agree, and strongly Agree. Strong internal consistency for APAS was established in national studies from several countries, including Poland [28], Macedonia [29], Turkey [30], and Lithuania [32].

### 2.4. Ethics Approval

All research procedures were conducted with strict adherence to ethical principles as set forth by the universities involved. Ethical approval was obtained from the Ethics Committee of the authors’ respective universities. Participants took part voluntarily and signed informed consent forms and parental written informed consents were obtained.

### 2.5. Statistical Analysis

The Statistical Package for the Social Sciences (SPSS, version 21) was used for data analyses. Data from the eight countries were pooled after cleaning and matching pre- and post-test data. Descriptive statistics were used to describe the student characteristics (means ± standard deviations). Confirmatory factor analysis was conducted using the Mplus software [35], version 8, to verify the measured variables representing the different constructs established for this study. Model goodness-of-fit was considered adequate when the comparative fit index (CFI) was ≥0.95, Tucker–Lewis Index (TLI) was ≥0.95, root mean square error of approximation (RMSEA) was <0.06, and the standardized path coefficients were ≥0.40 and statistically significant (*t*-values > 1.96). Based on results of the confirmatory factor analysis, variables were constructed by taking item means for each variable. Body mass index (BMI) scores of the participants were also computed. Comparability of the control and experimental groups at pre-test was ascertained using an independent sample *t*-test. Variable pre-test to post-test changes were evaluated using repeated measures analysis of variance (ANOVA) to determine time and time-by-group differences. Statistical significance was set at *p* < 0.05. Effect sizes of significant differences were evaluated using partial eta-squared (η^2^). Values of partial η^2^ equal to 0.0099, 0.0588, and 0.1379 were used as benchmarks for small, medium, and large effects [36].

## 3. Results

Of the 3053 participants at pre-test, 3036 provided gender information, and 2923 completed post-test evaluation, giving an attrition rate of 4.3% (= 130 of 3053). The attrition rate did not differ significantly between the control (2.9%) and experimental (5.1%) groups. Characteristics of the participants at pre-test are presented in Table 2. A slightly greater number of grade 3 (36.9%) than grade 5 (29.4%) students were in the sample, but student grade distributions were similar for the control and experimental groups. Independent sample *t*-tests showed no statistically significant difference between the control and experimental groups at pre-test in terms of age, body weight, body height, and participants’ attitudes toward PA as measured by the APAS questionnaire. The flow of participants through the study is presented in the Supplementary Figure S1.

Confirmatory factor analysis of the items showed a good fit for the APAS model according to the goodness-of-fit criteria with CFI equal to 0.964, TLI equal to 0.963, RMSEA equal to 0.039 (90% confidence interval = 0.038–0.039). All standardized path coefficients were in the range of 0.580 and 0.895 and statistically significant (*t*-values > 1.96).

Repeated measures of ANOVA identified a significant time-by-group interaction effect for BMI and all APAS variables except fitness. Significance was found for the APAS variables to include: self-efficacy with a large effect size; learning with a medium effect size; and benefits, importance, personal best, and fun with a small effect size (Table 3). Turkey’s post hoc test revealed significant differences between the control and experimental groups for self-efficacy, learning, benefits, importance, personal best, and fun at the post-test (significance order is largest to smallest).

**Figure 1:**
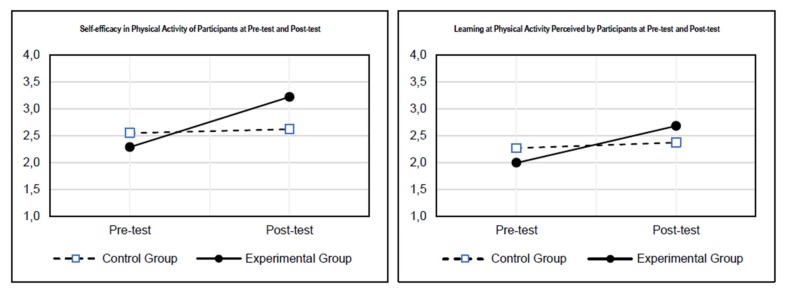

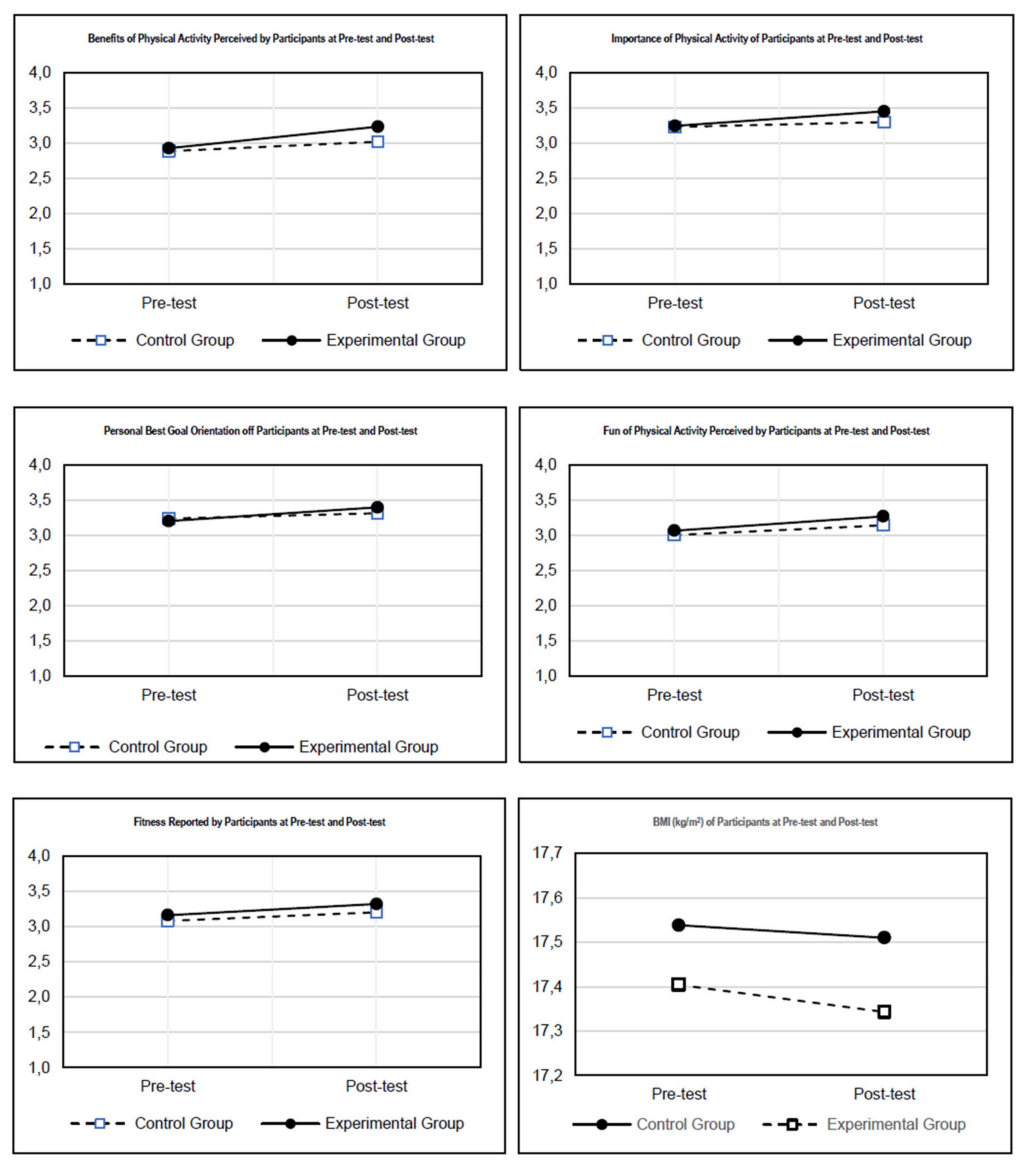
Distribution of the APAS scales’ means for the control and experimental groups (pre-test and post-test). Significant differences (* *p* ≤ 0.05) were found between the control and experimental groups for self-efficacy, learning, benefits, importance, personal best, and fun at the post-test (significance order is largest to smallest).

As presented in Figure 1, the experimental group had significant increases in attitudes toward PA from pre-test to post-test for self-efficacy and learning when compared to the control group. Although to a smaller extent, the same was observed for benefits and importance of PA.

## 4. Discussion

The purpose of the study was to evaluate the effectiveness of Brain Breaks® Physical Activity Solutions in changing attitudes toward PA in elementary students from eight countries. The study’s hypothesis was confirmed: regular participation in classroom PA breaks positively affected student attitudes toward PA via improvements in six of the seven APAS variables. Classroom-based exercise break videos improved the perceived PA benefits and importance, learning from the videos, self-efficacy in using exercise videos, increased interest in doing PA, and improved orientation toward personal best goals. Findings from this study are supported by similar studies where the effectiveness of Brain Breaks® videos was evaluated [24,28,29,30,32]. However, the results from this study differed from other studies when considering self-efficacy. For example, in Macedonia [29], Turkey [30], and Lithuania [32], most but not all APAS variables were improved after the intervention. While the experimental groups from the Turkey and Lithuania studies significantly improved all variables after intervention, the Macedonian study found significant effects for self-efficacy in the areas of learning, knowledge, and self-awareness. In Poland [28], the Brain Breaks® intervention contributed only to greater self-efficacy in the area of learning.

This study confirmed the positive impact that exercise videos have on learning. Education is a continuous process, involving the interchange of curricular subject matter during classes with non-curricular or informal educational activities during recess or PA periods [30]. Providing short PA breaks during the school day may not improve all areas of health [37]. The lack of improvement in fitness and BMI might be attributed to the teacher’s attitude toward specific exercise videos. In this regard, some teachers might focus on cognitive-related videos, while other teachers may focus on videos with low exercise intensity. In addition, the lack of significant improvement in fitness and BMI could also be attributed to the amount of time and intensity of movement during exercise videos. Data by Donnelly et al. [38] is, in part, supportive of our results with no significant change for BMI in children after three years of using the Physical Activity Across the Curriculum program.

The experimental group in the present study had significant gains in self-efficacy. Sun and Gao [39], in a randomized control trial, reported that active educational video games provided a more enjoyable learning experience when sufficient PA occurred. The improved self-efficacy due to the PA breaks without any changes in the academic curriculum and minimal interruptions in daily classroom management is an important finding. Emphasis should be placed on the importance that Brain Breaks® Physical Activity Solutions are not just forms of active breaks; rather, these breaks also provide learning and teaching strategies that are effective in promoting holistic learning [29,40].

Classroom PA breaks require little additional teacher preparation time, are enjoyable for students, result in positive classroom outcomes, and are acceptable teaching methods. Nonetheless, previous findings suggest that classroom-based PA is not always perceived positively by teachers and students [37]. In order to properly implement classroom-based PA breaks, teachers should consider barriers that students must overcome. Whitt-Glover et al. [37] reported that to reduce barriers and difficulties in classroom management, teachers should maintain flexibility within the classroom in choosing content, delivery, and making the activities enjoyable. Brain Breaks® activities offer an attractive choice for supplementing teaching methods and learning strategies while providing enjoyment.

This study is the first involving children from eight countries using a classroom-based PA intervention. However, a study limitation is the self-reporting nature of the questionnaire surveys. Intervention results might have been differently impacted if students’ PA levels were objectively measured. Although data from eight countries were analyzed in this study, we did not conduct differences analysis by country. Additionally, no consideration was given for educational (e.g., the number of PE hours in the different countries), sociocultural (e.g., involvement in sports activities during free time), and environmental (e.g., safe neighborhood) influences that might have affected the engagement and efficacy of the program in different populations.

## 5. Conclusions

The results of this study indicate that a four-month intervention of Brain Breaks® activities lead to improved student attitudes toward PA, perceived PA benefits, perception of importance of PA, enhanced learning, self-efficacy in using exercise videos, increased student interest in doing PA, and improved orientation of personal best goals when engaging in PA. The most important contribution of PA breaks in classroom settings an improved attitude toward physical health and general education of primary school children. Improving children’s attitudes toward PA is important for overall health and promoting sustainable social development. Using exercise videos during PA breaks is recommended as an interactive technology-based PA solution that is easily integrated into the school setting. Promoting classroom physical activity breaks is an effective approach for communicating the health benefits of physical activity. Additional studies in diverse populations are still needed to replicate our findings.

## Figures and Tables

**Table 1:** Sample subject distribution.

Grade	Control Group			Experimental Group		
	Number of Classes	Number of Males	Number of Females	Number of Classes	Number of Males	Number of Females
Grade 3	18	204	221	26	343	352
Grade 4	16	159	218	23	323	322
Grade 5	15	175	145	22	292	282
Total	49	538	584	71	958	956

Note: Only students who specified their gender are included in this Table.

**Table 2:** General characteristics of the participants at pre-test (*N* = 3036). a) Number of participants by gender, grade and country in the control and experimental groups.

Country	Grade	Control Group *n* = 1122 (37.0%)		Experimental Group *n* = 1914 (63.0%)		Number of Participants by Grade and Country
		Male	Female	Male	Female	
Croatia	3	13 (52.0%)	12 (48.0%)	37 (55.2%)	30 (44.8%)	92
	4	10 (40.0%)	15 (60.0%)	37 (55.2%)	36 (49.3%)	98
	5	15 (50.0%)	15 (50.0%)	32 (50.8%)	31 (49.2%)	93
Lithuania	3	8 (33.3%)	16 (66.7%)	15 (57.7%)	11 (42.3%)	50
	4	11 (42.3%)	15 (57.5%)	8 (33.3%)	16 (66.7%)	50
Macedonia	3	24 (53.3%)	21 (46.7%)	31 (59.6%)	21 (40.4%)	97
	4	26 (52.0%)	24 (48.0%)	32 (58.2%)	23 (41.8%)	105
	5	19 (52.8%)	17 (47.2%)	23 (51.1%)	22 (48.9%)	81
Poland	3	10 (50.0%)	10 (50.0%)	51 (46.8%)	58 (53.2%)	129
	4	12 (60.0%)	8 (40.0%)	59 (51.8%)	55 (48.2%)	134
	5	16 (64.0%)	9 (36.0%)	53 (48.6%)	56 (51.4%)	134
Romania	3	22 (45.8%)	26 (54.2%)	42 (45.2%)	51 (54.8%)	141
	4	13 (31.7%)	28 (68.3%)	43 (50.0%)	43 (50.0%)	127
	5	13 (56.5%)	10 (43.5%)	42 (47.2%)	47 (52.8%)	112
Serbia	3	49 (54.4%)	41 (45.6%)	44 (50.0%)	44 (50.0%)	178
	4	32 (48.5%)	34 (51.5%)	33 (54.1%)	28 (45.9%)	127
	5	26 (51.0%)	25 (49.0%)	31 (58.5%)	22 (41.5%)	104
South Africa	3	40 (44.9%)	49 (55.1%)	41 (40.2%)	61 (59.8%)	191
	4	32 (34.8%)	60 (65.2%)	49 (48.5%)	52 (51.5%)	193
	5	55 (55.6%)	44 (44.4%)	56 (55.4%)	45 (44.6%)	200
Turkey	3	38 (45.2%)	46 (54.8%)	82 (51.9%)	76 (48.1%)	242
	4	23 (40.4%)	34 (59.6%)	62 (47.3%)	69 (52.7%)	188
	5	31 (55.4%)	25 (44.6%)	55 (48.2%)	59 (51.8%)	170
All countries	3	204 (48.0%)	221 (52.0%)	343 (49.4%)	352 (50.6%)	1120
	4	159 (42.2%)	218 (57.8%)	323 (50.1%)	322 (49.9%)	1022
	5	175 (54.7%)	145 (45.3%)	292 (50.9%)	282 (49.1%)	894
	All	538 (48.0%)	584 (52.0%)	958 (50.1%)	956 (49.9%)	3036

Note: Figures with brackets are percentages within gender, grade level, and country.

**Table 3:** Descriptive statistics of the control and experimental groups and results of the repeated measures ANOVA.

Variables	Groups	Pre-Test	Post-Test	Time		Time × Group	
		M ± SD	M ± SD	F (*p*)	η^2^	F (*p*)	η^2^
Benefits	CON	2.888 ± 0.650	3.021 ± 0.619	346.518 **	0.107	53.175 **	0.018
	EXP	2.929 ± 0.691	3.235 ± 0.538				
Importance	CON	3.231 ± 0.634	3.298 ± 0.589	159.169 **	0.052	40.564 **	0.014
	EXP	3.246 ± 0.659	3.452 ± 0.536				
Learning	CON	2.268 ± 0.709	2.374 ± 0.748	443.503 **	0.177	236.484 **	0.103
	EXP	1.997 ± 0.827	2.681 ± 0.696				
Self-efficacy	CON	2.550 ± 0.822	2.619 ± 0.847	493.599 **	0.186	366.258 **	0.145
	EXP	2.288 ± 0.997	3.219 ± 0.624				
Fun	CON	3.008 ± 0.673	3.143 ± 0.616	224.662 **	0.074	9.227 **	0.003
	EXP	3.068 ± 0.686	3.271 ± 0.593				
Fitness	CON	3.081 ± 0.715	3.206 ± 0.658	151.520 **	0.050	2.066 (ns)	
	EXP	3.164 ± 0.692	3.322 ± 0.590				
Personal best	CON	3.237 ± 0.771	3.315 ± 0.732	137.790 **	0.045	25.530 **	0.009
	EXP	3.203 ± 0.811	3.399 ± 0.658				
BMI (kg/m^2^)	CON	17.538 ± 2.723	17.510 ± 2.582	5.782 *	0.002	0.801 (ns)	
	EXP	17.404 ± 2.562	17.343 ± 2.453				

CON: Control group, EXP: Experimental group; M: Mean; SD: Standard deviation; * *p* < 0.05, ** *p* < 0.01, ns: not significant at 0.05 Note: In total, there were 3053 students (the control groups had *n* = 1137 and the experimental groups had *n* = 1916).

## Supplementary Materials

The following are available online at https://www.mdpi.com/1660-4601/17/5/1666/s1, Figure S1: Flow diagram, Table S1: Distribution of Students in the Control and Experimental Groups.

## References

[1] Kuan G., Rizal H., Hajar M.S., Chin M.K., Mok M.M.C. (2019). Bright sports, physical activity investments that work: Implementing brain breaks in Malaysian primary schools. Br. J. Sports Med..

[2] Wahid A., Manek N., Nichols M., Kelly P., Foster C., Webster P., Kaur A., Friedemann Smith C., Wilkins E., Rayner M. (2016). Quantifying the association between physical activity and cardiovascular disease and diabetes: A systematic review and meta-analysis. J. Am. Heart Assoc..

[3] World Health Organization (WHO) (2018). Phys. Act. http://www.who.int/news-room/fact-sheets/detail/physical-activity.

[4] Turkish Ministry of Health (2014). Turkey Nutrition and Health Survey 2010-Evaluation of Nutritional Status and Habits Report. General Directorate of Health Research, Ankara. https://www.saglikaktuel.com/d/file/tbsa_beslenme_yayini.pdf..

[5] Djordjic V., Radisavljevic S., Milanovic I., Bozic P., Grbic M., Jorga J. (2016). WHO European Childhood Obesity Surveillance Initiative in Serbia: A prevalence of overweight and obesity among 6-9-year-old school children. J. Pediatr. Endocrinol. Metab..

[6] Rossouw H.A., Grant C.C., Viljoen M. (2012). Overweight and obesity in children and adolescents: The South African problem. South Afr. J. Sci..

[7] Chirita-Emandi A., Barbu C.G., Cinteza E.E., Chesaru B.I., Gafencu M., Mocanu V., Pascanu I.M., Tatar S.A., Balgradean M., Dobre M. (2016). Overweight and underweight prevalence trends in children from Romania—Pooled analysis of cross-sectional studies between 2006 and 2015. Obes. Facts.

[8] Robinson L.E., Stodden D.F., Barnett L.M., Lopes V.P., Logan S.W., Rodrigues L.P., D’Hondt E. (2015). Motor competence and its effect on positive developmental trajectories of health. Sports Med..

[9] Donnelly J.E., Hillman C.H., Greene J.L., Hansen D.M., Gibson C.A., Sullivan D.K., Poggio J., Mayo M.S., Lambourne K., Szabo-Reed A.N. (2017). Physical activity and academic achievement across the curriculum: Results from a 3-year cluster-randomized trial. Prev. Med..

[10] Hillman C.H., Erickson K.I., Hatfield B.D. (2017). Run for your life! Childhood physical activity effects on brain and cognition. Kinesiol. Rev..

[11] Schmidt M., Benzing V., Kamer M. (2016). Classroom-based physical activity breaks and children’s attention: Cognitive engagement works!. Front. Psychol..

[12] Donnelly J.E., Hillman C.H., Castelli D., Etnier J.L., Lee S., Tomporowski P., Lambourne K., Szabo-Reed A.N. (2016). Physical activity, fitness, cognitive function, and academic achievement in children: A systematic review. Med. Sci. Sports Exerc..

[13] Mullender-Wijnsma M.J., Hartman E., de Greeff J.W., Bosker R.J., Doolaard S., Visscher C. (2015). Improving academic performance of school-age children by physical activity in the classroom: 1-year program evaluation. J. Sch. Health.

[14] Watson A., Timperio A., Brown H., Best K., Hesketh K.D. (2017). Effect of classroom-based physical activity interventions on academic and physical activity outcomes: A systematic review and meta-analysis. Int. J. Behav. Nutr. Phys. Act..

[15] Kumar S., Kelly A.S. (2017). Review of childhood obesity: From epidemiology, etiology, and comorbidities to clinical assessment and treatment. Mayo Clin. Proc..

[16] Janssen I., LeBlanc A.G. (2010). Systematic review of the health benefits of physical activity and fitness in school-aged children and youth. Int. J. Behav. Nutr. Phys. Act..

[17] Brindova D., Veselska Z.D., Klein D., Hamrik Z., Sigmundova D., Van Dijk J.P., Reijneveld S.A., Madarasova Geckova A. (2014). Is the association between screen-based behaviour and health complaints among adolescents moderated by physical activity?. Int. J. Public Health.

[18] Tremblay M.S., Barnes J.D., González S.A., Katzmarzyk P.T., Onywera V.O., Reilly J., Tomkinson G.R., Global Matrix 2.0 Research Team (2016). Global Matrix 2.0: Report card grades on the physical activity of children and youth comparing 38 countries. J. Phys. Act. Health.

[19] Webster C.A., Russ L., Vazou S., Goh T.L., Erwin H. (2015). Integrating movement in academic classrooms: Understanding, applying and advancing the knowledge base. Obes. Rev..

[20] Hills A.P., Dengel D.R., Lubans D.R. (2015). Supporting public health priorities: Recommendations for physical education and physical activity promotion in schools. Prog. Cardiovasc. Dis..

[21] Arundell L., Fletcher E., Salmon J., Veitch J., Hinkley T. (2016). A systematic review of the prevalence of sedentary behavior during the after-school period among children aged 5–18 years. Int. J. Behav. Nutr. Phys. Act..

[22] Trudeau F., Shephard R.J. (2008). Physical education, school physical activity, school sports and academic performance. Int. J. Behav. Nutr. Phys. Act..

[23] Reis R.S., Salvo D., Ogilvie D., Lambert E.V., Goenka S., Brownson R.C. (2016). Lancet Physical Activity Series 2 Executive Committee. Scaling up physical activity interventions worldwide: Stepping up to larger and smarter approaches to get people moving. Lancet.

[24] Rasberry C.N., Lee S.M., Robin L., Laris B.A., Russell L.A., Coyle K.K., Nihiser A.J. (2011). The association between school-based physical activity, including physical education, and academic performance: A systematic review of the literature. Prev. Med..

[25] Podnar H., Novak D., Radman I. (2018). Effects of a 5-minute classroom-based physical activity classroom-based physical activity on on-task behavior and physical activity levels. Kinesiol. Int. J. Fundam. Appl. Kinesiol..

[26] McMullen J., Kulinna P., Cothran D. (2014). Physical activity opportunities during the school day: Classroom teachers’ perceptions of using activity breaks in the classroom. J. Teach. Phys. Educ..

[27] Edginton C.R., Chin M.K., Demirhan G., Asci H., Bulca Y., Erturan-Ogut E. (2016). Global forum for physical education pedagogy 2016—Technology, networking and best practice in physical education and health: Local to global. Int. J. Phys. Educ. A Rev. Publ..

[28] Müller A.M., Khoo S. (2016). Interdisciplinary, child-centered collaboration could increase the success of potentially successful Internet-based physical activity interventions. Acta Paediatr..

[29] Glapa A., Grzesiak J., Laudanska-Krzeminska I., Chin M.K., Edginton C.R., Mok M.M.C., Bronikowski M. (2018). The Impact of Brain Breaks Classroom-Based Physical Activities on Attitudes toward Physical Activity in Polish School Children in Third to Fifth Grade. Int. J. Environ. Res. Public Health.

[30] Popeska B., Jovanova-Mitkovska S., Chin M.K., Edginton C.R., Mok M.M.C., Gontarev S. (2018). Implementation of Brain Breaks^®^ in the Classroom and Effects on Attitudes toward Physical Activity in a Macedonian School Setting. Int. J. Environ. Res. Public Health.

[31] Uzunoz F.S., Chin M.K., Mok M.M.C., Edginton C.R., Podnar H., Dumon D., Hofmann A.R., Diketmuller R., Koenen K., Bailey R., Zinkler C. (2017). The effects of technology supported brain breaks on physical activity in school children. Passionately Inclusive: Towards Participation and Friendship in Sport: Festschrift für Gudrun Doll-Tepper.

[32] Martin A.J. (2006). Personal bests (PBs): A proposed multidimensional model and empirical analysis. Br. J. Educ. Psychol..

[33] HOPSports Website (2014). Interactive Youth Physical Education Training System. http://www.hopsports.com.

[34] Mok M.M.C., Chin M.K., Chen S., Emeljanovas A., Mieziene B., Bronikowski M., Laudanska-Krzeminska I., Milanovic I., Pasic M., Balasekaran G. (2015). The psychometric properties of the Attitudes toward Physical Activity Scale: A Rasch analysis based on data from five locations. J. Appl. Meas..

[35] Emeljanovas A., Mieziene B., Mok M.M.C., Chin M.K., Cesnaitiene V.J., Fatkulina N., Trinkuniene L., Lopez Sanchez G.F., Diaz Suarez A. (2018). The effect of an interactive program during school breaks on attitudes toward physical activity in primary school children. Ann. Psychol..

[36] Muthén L.K., Muthén B.O. (1998). Mplus User’s Guide.

[37] Richardson J.T. (2011). Eta squared and partial eta squared as measures of effect size in educational research. Educ. Res. Rev..

[38] Whitt-Glover M.C., Port A.T. (2013). Do Short Physical Activity Breaks in Classrooms Work? Robert Wood Johnson Foundation. https://activelivingresearch.org/sites/activelivingresearch.org/files/ALR_Brief_ActivityBreaks_Feb2013.pdf.

[39] Donnelly J.E., Greene J.L., Gibson C.A., Smith B.K., Washburn R.A., Sullivan D.K., DuBose K., Mayo M.S., Schmelzle K.H., Ryan J.J. (2009). Physical Activity Across the Curriculum (PAAC): A randomized controlled trial to promote physical activity and diminish overweight and obesity in elementary school children. Prev. Med..

[40] Sun H., Gao Y. (2016). Impact of an active educational video game on children’s motivation, science knowledge, and physical activity. J. Sport Health Sci..

